# Evaluation of Phototoxicity of Short-Wavelength Laser Light Utilizing PCNA Accumulation

**DOI:** 10.3390/mi15050646

**Published:** 2024-05-13

**Authors:** Tetsuya Matsuyama, Noboru Osaka, Mikiya Yamaguchi, Naohiro Kanamaru, Kenji Wada, Ai Kawakita, Kaori Murata, Kenji Sugimoto, Koichi Okamoto

**Affiliations:** 1 Graduate School of Engineering, Osaka Metropolitan University, 1-1 Gakuen-cho, Naka-ku, Sakai 599-8531, Osaka, Japan; 2 Graduate School of Agriculture, Osaka Metropolitan University, 1-1 Gakuen-cho, Naka-ku, Sakai 599-8531, Osaka, Japan

**Keywords:** phototoxicity, quantitative evaluation, live cell imaging, proliferating cell nuclear antigen (PCNA), short-wavelength laser light, cell viability

## Abstract

In recent years, diseases such as age-related macular degeneration and retinal pigment degeneration caused by excessive exposure to short-wavelength visible light have become significant concerns. With the aim of quantitatively evaluating the toxicity of short-wavelength light, proliferating cell nuclear antigen (PCNA) accumulation at the irradiation site was investigated using live cell imaging techniques to irradiate individual living cells with short-wavelength laser light. By examining the dependency of PCNA accumulation on the irradiation site within the cells and their cell cycle, it was observed that PCNA accumulation occurred only when the cell nucleus of cells in the S phase of the cell cycle was irradiated. We investigated the accumulation of PCNA at the laser irradiation site using laser light at wavelengths of 405 nm and 375 nm, with intensities ranging from 0.5 μW to 9.0 μW. The results confirmed an increase in PCNA accumulation with increasing intensity, and a higher accumulation was observed with laser light irradiation at a wavelength of 375 nm compared to 405 nm. By comparing the PCNA accumulation and 24 h cell viability, we demonstrated the feasibility of quantitatively assessing laser light toxicity through the measurement of PCNA accumulation.

## 1. Introduction

In recent years, with the widespread adoption of various semiconductor optical devices such as white LEDs for illumination, liquid crystal displays, and smartphones, exposure to short-wavelength visible light in daily life has increased significantly. Diseases such as circadian rhythm disruption and insomnia [1,2], age-related macular degeneration [3,4,5], and retinal pigment degeneration [6,7], resulting from excessive exposure to large amounts of short-wavelength visible light, have become major concerns. It has been reported that exposure of cells to short-wavelength visible light induces apoptosis and necroptosis [8,9,10,11,12,13,14]. After the pandemic of novel coronavirus infections, the induction of cell death by short-wavelength visible light irradiation has attracted attention in medical fields such as sterilization, antibacterials, and disease treatment [15,16,17,18].

Ultraviolet (UV) radiation, categorized into UVA (320–400 nm), UVB (280–320 nm), and UVC (100–280 nm), is well-known for causing DNA damage through direct and indirect mechanisms [19,20,21,22,23]. Threshold limit values (TLVs) for UV exposure have been established by organizations such as the American Conference of Governmental Industrial Hygienists (ACGIH) [19]. UVB and UVC are directly absorbed by DNA, leading to DNA lesions by the formation of cyclobutane pyrimidine dimers (CPDs), which have been elucidated through methods like DNA sequencing [20,21]. While UVA is not directly absorbed by DNA, it mainly generates reactive oxygen species (ROS) through light-absorbing substances, causing oxidative damage to DNA, as evidenced by cell survival assays and immunological assays [22,23]. Short-wavelength visible light, similar to UVA, is believed to cause indirect DNA damage [24,25,26] since it is not directly absorbed by DNA. However, details such as wavelength dependency are not fully understood, necessitating further investigation of its mechanisms. Previous studies on phototoxicity have primarily irradiated LED light over entire cell culture dishes and observed only cell viability through cell survival assay [8,9,10,11,12]. Experiments conducted in test tubes may not accurately represent cellular dynamics due to differences in oxygen concentration. Therefore, evaluating phototoxicity in living cells by observing cell dynamics immediately after irradiation is necessary for a clearer understanding. To address this, utilizing a live cell imaging technique, we have proposed criteria for determining cell death based on changes in nuclear area and performed phototoxicity evaluations on living cells by combining a laser diode [27]. Live cell imaging is a cell observation technique that allows real-time observation of living cells by introducing fluorescent proteins or quantum dots into specific sites of the target cell and capturing the fluorescence generated by irradiating the excitation light [28,29,30]. Unlike test tube environments used for enzyme reaction evaluations, live cell imaging allows toxicity assessments in environments closer to living organisms. By adjusting the concentration of fluorescent dyes and proteins, as well as the intensity of the excitation light, living cells can be observed without toxicity induced by the excitation light. Additionally, by introducing multiple fluorescent dyes or proteins into cells, it becomes possible to visualize and observe multiple parts of the cell simultaneously. This enables the individual visualization of cellular structures and allows for the observation of the dynamics of living cells while identifying specific intracellular structures. The tracking of individual cells by live cell imaging makes it possible to observe not only the result of cell death but also the process of damage through changes in cell dynamics. Additionally, using laser light enables the selection of irradiation sites on cells and sufficient irradiation with a short exposure time, allowing for irradiation of cells in a specific phase of the cell cycle. In this study, we irradiated living cells in specific phase of the cell cycle with short-wavelength laser light and examined the accumulation of PCNA and cell viability to investigate the phototoxicity of short-wavelength visible light.

## 2. Methods

**Figure 1:**
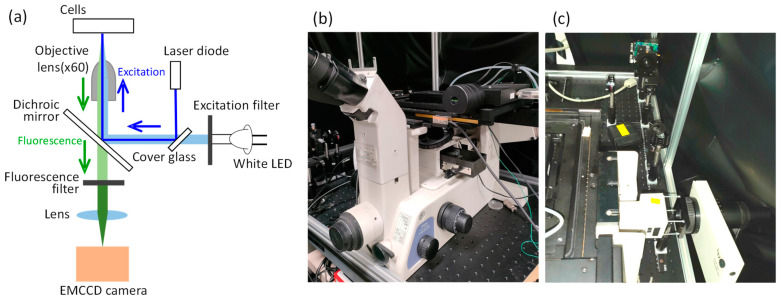
(**a**) Schematic diagram of experimental setup. Photograph of (**b**) microscope and (**c**) illumination optical system.

In this study, human malignant-melanoma-derived cells (MDA-MB-435S) were utilized, with the cell nucleus visualized using the red fluorescent protein mPlum-histone H3 and the proliferating cell nuclear antigen (PCNA) visualized using the green fluorescent protein EGFP [29]. The two-color fluorescent cells used in this study were constructed by co-authors, and the details of the creation process are same to those described in the Refs. [29,31]. These cells were cultured in glass bottom dishes (Iwaki 3000-035, AGC Techno Glass Co., Ltd., Shizuoka, Japan) using Dulbecco’s modified Eagle medium (DMEM, Nissui, Nissui Pharmaceutical Co., Ltd., Tokyo, Japan) with 10% fetal bovine serum (Biowest, Nuaille, France) under conditions of 37 °C and 5% CO_2_. The schematic diagram of the experimental setup is illustrated in Figure 1a. Photographs of the microscope and optical system are shown in Figure 1b,c, respectively. The optical system of the fluorescence microscope (TE-300, Nikon Corp., Tokyo, Japan) consisted of a white LED (MWWHL3, THORLABS JAPAN Inc., Tokyo, Japan), an oil-immersion objective lens (PlanApo VC 60X, Nikon Corp., Tokyo, Japan), excitation and fluorescence filters, a dichroic mirror (Chroma Technology Corp., Bellows Falls, VT, USA), a stage top incubator (INU-ZILCS-F1, Tokai Hit Co. Ltd., Shizuoka, Japan), and an EMCCD camera (ORCA-ER, Hamamatsu Photonics K. K., Shizuoka, Japan). The excitation light was reflected by the dichroic mirror and irradiated onto the cells in the dish using the oil-immersion objective lens. The fluorescence emitted from the fluorescent proteins within living cells passed through the objective lens and the dichroic mirror, was separated from the excitation light using a fluorescence filter, and detected by the EMCCD camera. The transmission wavelength ranges of the excitation/fluorescence filters used were 525 ± 30 nm/630 ± 60 nm for mPlum measurements and 425 ± 25 nm/520 ± 20 nm for EGFP measurements. Semiconductor lasers with wavelengths of 375 nm and 405 nm were controlled using a current controller (LDC201C, THORLABS JAPAN Inc., Tokyo, Japan) and a temperature controller (TED200C, THORLABS JAPAN Inc., Tokyo, Japan). After converting the light into parallel beams using an objective lens, the intensity was adjusted by an attenuator. The laser light was introduced into the microscope using the surface reflection (approximately 6%) of a cover glass inserted into the LED illumination system at a 45-degree angle, and specific sites within cells were illuminated by focusing with the objective lens. The laser spot diameter at the focal point was observed to be approximately 2.0 µm, thus, irradiating with a laser intensity of 3.0 µW corresponds to an irradiance of approximately 100 W/cm^2^. To minimize potential damage to living cells caused by LED excitation light during fluorescence observation, LED light was only irradiated for 2.4 s every minute during EGFP observation and for 2.4 s every 5 min during mPlum observation to capture images. Initially, observations of living cells were conducted without irradiating laser light to confirm clear observation of the dynamics of the cell nucleus and PCNA. Subsequently, short-wavelength laser light was irradiated, and detailed investigations were conducted on PCNA accumulation at the site of laser light irradiation within cells.

## 3. Results and Discussion

### 3.1. Cell Observation via Live Cell Imaging

**Figure 2:**
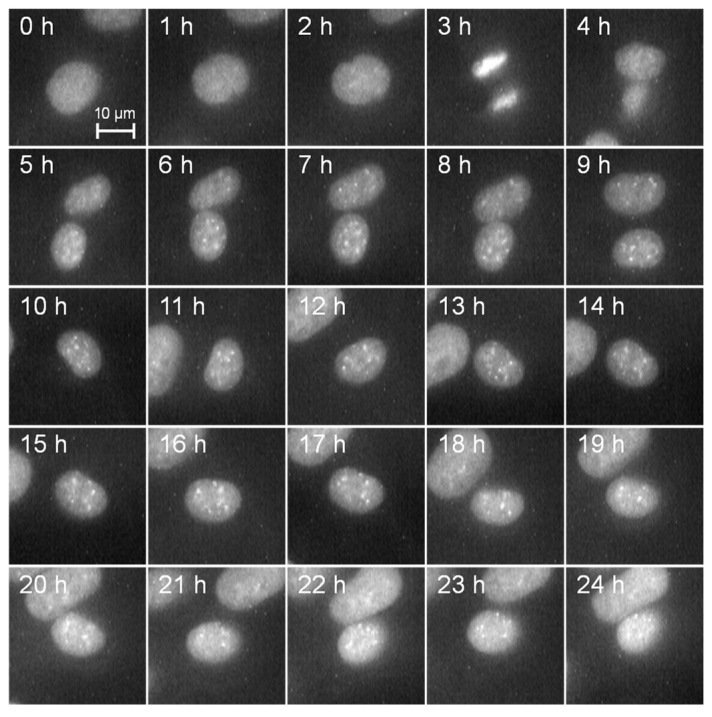
Time-lapse images of mPlum-histone H3 in MDA-MB-435S cells over 24 h. The cell nucleus was clearly observed over 24 h by measuring the fluorescence from mPlum.

The dynamics of the cell nucleus were observed every hour for 24 h by fluorescence observation from mPlum without irradiating short-wavelength laser light, as depicted in Figure 2. The scale bar corresponds to 10 µm. It can be clearly observed that the cell nucleus labeled using mPlum remains distinct without fading throughout the entire observation period. Furthermore, it is evident from the observation that normal cell division occurs three hours after the start of observation, indicating the feasibility of dynamic cell observation.

**Figure 3:**
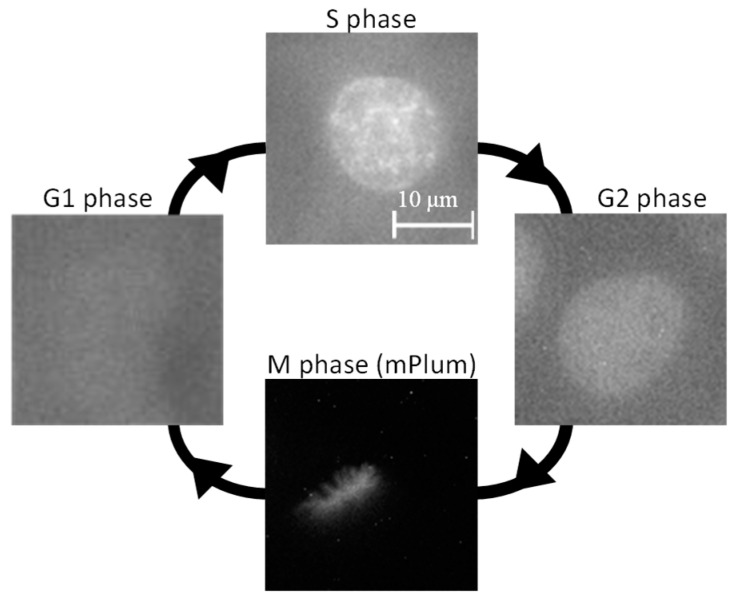
PCNA distributions in each phase of cell cycle. PCNA diffuses throughout the cytoplasm during G1 phase of cell cycle, with no distinct visualization of the cell nucleus. In the S phase of the cell cycle, PCNA translocates into the nucleus and exhibits dot-like localization. Subsequently, during the G2 and M phases of the cell cycle, PCNA disperses back into the cytoplasm. M phase of cell cycle could be distinguished by observing aligned thread-like chromosomes.

PCNA, labeled with EGFP, is a nuclear protein involved in DNA repair, replication, and cell cycle regulation [32,33], primarily localizing within the cell nucleus during the active DNA replication phase, known as the S phase [34,35,36]. Therefore, by visualizing the dynamics of PCNA, it is possible to distinguish different phases of the cell cycle. The results of PCNA dynamics observation are shown in Figure 3. As depicted in Figure 3, during the G1 phase of the cell cycle, PCNA diffuses throughout the cytoplasm [32,33], resulting in no distinct visualization of the cell nucleus. However, in the S phase of the cell cycle, PCNA translocates into the nucleus and exhibits dot-like localization. Subsequently, it disperses back into the cytoplasm during the G2 and M phases of the cell cycle. Additionally, the M phase can be distinguished by observing aligned thread-like chromosomes in the mPlum fluorescence images. Thus, by monitoring PCNA distribution, it is possible to identify the cell cycle, enabling the irradiation of specific cell sites with laser light in a particular phase of the cell cycle. In this study, cells in the S and G1 phases, characterized by the dot-like distribution of PCNA and the presence of smaller daughter cell pairs immediately post-cell division, were chosen for investigating the effects of laser irradiation.

### 3.2. Accumulation of PCNA by Laser Irradiation

PCNA is a nuclear protein involved in DNA repair, replication, and cell cycle regulation. It is believed that when indirect DNA damage occurs due to laser irradiation, PCNA accumulates at the site of DNA damage. Therefore, by irradiating laser light onto live cells and quantitatively evaluating the degree of PCNA accumulation at the irradiation site, we estimated the extent of DNA damage. When DNA damage is induced by laser irradiation, the accumulation of PCNA typically occurs within a short time after laser irradiation. This implies that the effects of laser irradiation on cells can be rapidly assessed without determining cell death after a time equivalent to the cell cycle time has elapsed. Additionally, it is conceivable to quantitatively evaluate low-level damage that may not result in cell death.

**Figure 4:**
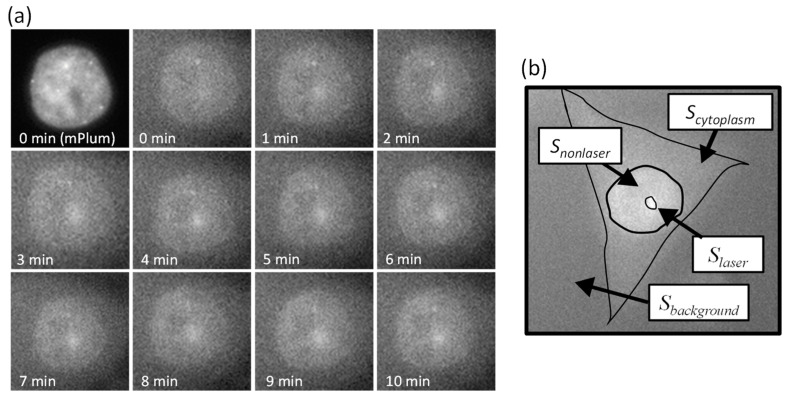
(**a**) PCNA accumulation in cell nucleus of MDA-MB-435S cell observed by 405 nm laser irradiation. An increase in EGFP fluorescence intensity was observed at the laser-irradiated site as time elapsed after laser irradiation. (**b**) Definition of PCNA accumulation by brightness of fluorescence image.

Cells in the S phase of the cell cycle were irradiated with laser light at a wavelength of 405 nm and intensity of 3 µW for 60 s, and the accumulation of PCNA at the laser irradiation site was observed for 10 min, as depicted in Figure 4a. Considering the saturation tendency of PCNA accumulation, the measurement time was set to 10 min. An increase in EGFP fluorescence intensity was observed at the laser-irradiated site as time elapsed after laser irradiation, confirming PCNA accumulation. Considering the function of PCNA, it can be assumed that DNA damage is induced at the site of laser irradiation.

To evaluate the degree of PCNA accumulation independently of individual variations such as the amount of introduced fluorescent proteins and the total amount of PCNA, PCNA accumulation was defined as the brightness ratio (BP) as follows:

(1) $${BP}_{nucleus/cytoplasm}=\frac{S_{laser}-S_{cytoplasm/background}}{S_{nonlaser}-S_{cytoplasm/background}},$$

where *S_laser_*, *S_nonlaser_*, *S_cytoplasm_*, and *S_background_* represent the average brightness of the laser-irradiated area within the cell nucleus or cytoplasm, the non-irradiated area within the same tissue, the cytoplasm, and the background, respectively. These values (pixel value) are obtained from PCNA observation images, as illustrated in Figure 4b. Using this equation, the influence of individual variations in each cell was mitigated, allowing for the quantitative evaluation of the degree of PCNA accumulation.

**Figure 5:**
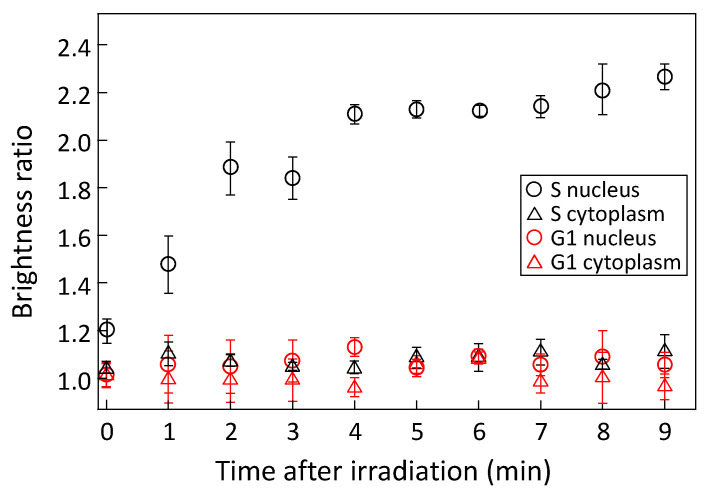
Temporal variation of brightness ratio after 405 nm laser irradiation to cell nucleus and cytoplasm in S and G1 phase. *p*-value < 0.00001 between S nucleus and other results.

Figure 5 shows the temporal variations of PCNA accumulation in the nucleus and cytoplasm of cells in the S and G1 phases of the cell cycle. The cells were irradiated with a 405 nm wavelength laser light at an intensity of 6.0 µW for 60 s, and PCNA accumulation was measured for 10 min after irradiation. Measurements were conducted on five or more cells for each condition, and the mean values and standard errors were calculated. When the cell nucleus in the S phase was irradiated with laser light, the accumulation of PCNA increased significantly over time after laser irradiation and saturated at approximately 2.2. PCNA aggregates within the cell nucleus during the S phase of the cell cycle for DNA repair and replication purposes, allowing it to rapidly accumulate at sites of DNA damage induced by laser irradiation. This suggests that cells in the S phase exhibit a high DNA repair capacity. Conversely, when the cell nucleus in the G1 phase was irradiated with laser light, no PCNA accumulation was observed. In cells in the G1 phase, which is the interval period between the M phase and the S phase, PCNA diffuses throughout the cell, making it difficult for PCNA to accumulate in the nucleus in response to DNA damage caused by laser irradiation. Additionally, regardless of the phase of the cell cycle, no PCNA accumulation was observed when the cytoplasm was irradiated with laser light. Since DNA is not present in the cytoplasm, PCNA does not contribute to repairing DNA damage caused by laser irradiation.

**Figure 6:**
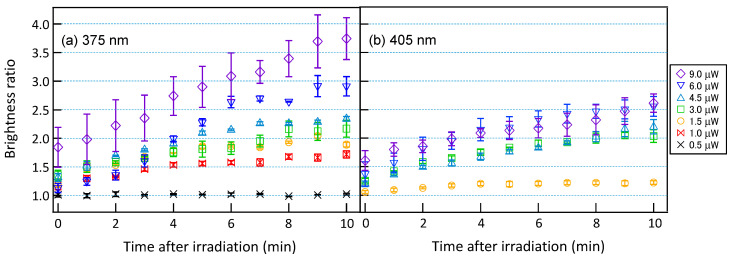
Temporal variation of brightness ratio after irradiation of (**a**) 375 nm and **(b**) 405 nm laser with various intensities to cell nuclei of MDA-MB-435S cells. *p*-value = 0.00016 between the results of 375 nm and 405 nm.

For cells in the S phase exhibiting significant PCNA accumulation, laser light at a wavelength of 405 nm was irradiated at intensities ranging from 1.5 to 9.0 µW, and laser light at a shorter wavelength of 375 nm, which is expected to have higher DNA damage capability, was irradiated at intensities ranging from 0.5 to 9.0 µW for 60 s. The temporal variations of PCNA accumulations were subsequently measured. Figure 6a shows the temporal variation of PCNA accumulation for irradiation of laser light with a wavelength of 375 nm, while Figure 6b shows the corresponding temporal variation for laser light with a wavelength of 405 nm. For laser light with a wavelength of 375 nm, PCNA accumulation saturated over time at approximately 1.0, 1.7, 2.0, 2.2, 2.4, 2.9, and 3.7, from the lowest to highest irradiance, indicating that as the irradiance increased, PCNA accumulation proportionally increased. Conversely, for laser light at a wavelength of 405 nm, PCNA accumulation saturated over time at approximately 1.2, 2.0, 2.2, 2.5, and 2.6, from the lowest to highest irradiance. At irradiance above 3.0 µW, the saturation level of PCNA accumulation asymptotically approached a consistent value of about 2.6 as the irradiance increased. In all laser light intensities, PCNA accumulation was higher for a wavelength of 375 nm compared to 405 nm, suggesting that the shorter wavelength of 375 nm has a more pronounced effect on DNA. Additionally, as the irradiation intensity increases, the saturation level of PCNA accumulation approaches an asymptotic value for laser light with a wavelength of 405 nm, whereas for laser light with a wavelength of 375 nm, the saturation level increases proportionally with the irradiation intensity. The differences in the saturation tendency with respect to irradiance may reflect variations in the impact on DNA depending on the wavelength of the irradiating laser light.

### 3.3. Comparison of PCNA Accumulation and Cell Survival Rate

To explore the relationship between PCNA accumulation induced by laser irradiation and actual cell viability, we conducted live cell imaging over 24 h, corresponding to the cell cycle time, after irradiation with laser light. In our previous research, we observed that exposure to short-wavelength visible light resulted in a gradual reduction in the area of the cell nucleus, followed by a rapid decrease in area and significant movement of the nucleus centroid, ultimately leading to cell death [27]. We also confirmed that when the cell nucleus area decreases to less than 80% of its pre-irradiation size, cell death occurs within 24 h thereafter without recovery. Therefore, in this study, cells with nuclear areas reduced to less than 80% of their pre-irradiation size or showing signs of apoptosis-like fragmentation and aggregation of DNA [37] were classified as dead.

**Figure 7:**
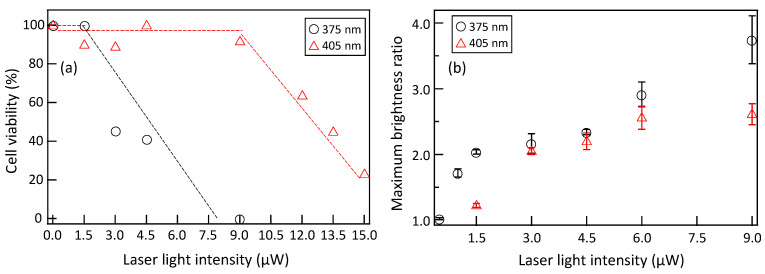
Dependence of (**a**) cell viability and (**b**) maximum brightness ratio on irradiated laser intensity. *p*-value = 0.031 for maximum brightness ratio.

We exposed cell nuclei to laser light with a wavelength of 405 nm, ranging in intensity from 1.5 to 15.0 µW, and laser light with a wavelength of 375 nm, ranging in intensity from 1.5 to 9.0 µW, for 60 s. The results of measuring the 24 h cell viability of 15 to 20 cells under each irradiation condition are shown in Figure 7a. Figure 7b shows the intensity dependence of the maximum PCNA accumulation obtained during the observation after laser irradiation, as obtained from Figure 6. For irradiation of laser light with a wavelength of 405 nm, cell death was not induced at intensities up to 9.0 µW, and the cell viability gradually decreased with increasing intensity beyond 12.0 µW. At low irradiation intensities below 9.0 µW, although saturation tendencies were observed, as shown in Figure 7b, sufficient PCNA accumulated at the irradiation site to prevent cell death, presumably facilitating DNA repair. Intensities above 12.0 µW led to the saturation of PCNA accumulation, resulting in insufficient DNA repair and subsequent cell death. By quantitatively evaluating PCNA accumulation, it becomes possible to assess the DNA damage, ranging from minor damage that does not result in cell death to severe damage that leads to cell death. In contrast, for laser light with a wavelength of 375 nm, cell death was not induced at an intensity of 1.5 µW, indicating that sufficient DNA repair occurred due to PCNA accumulation, preventing cell death. A significant decrease in cell viability was observed with increasing irradiation intensity from 3.0 µW, and at 9.0 µW, all cells showed cell death. Despite PCNA accumulation levels similar to those induced by 405 nm laser light, cell death occurred at a lower irradiance with 375 nm laser irradiation, indicating that the threshold of PCNA accumulation triggering cell death varies depending on the wavelength. Therefore, by specifying the wavelength, it is possible to quantitatively assess not only DNA damage but also cell death through PCNA accumulation. Comparing the irradiation intensities required to induce 50% cell death, laser light with a wavelength of 375 nm was estimated to have approximately 4.5 times higher phototoxicity than that of a wavelength of 405 nm.

## 4. Conclusions

In this study, with the aim of quantitatively evaluating the toxicity of short-wavelength visible light, we irradiated living cells with short-wavelength laser light using a live cell imaging technique and evaluated PCNA accumulation at the irradiated sites. We found that significant PCNA accumulation occurred only when the nucleus of cells in the S phase of the cell cycle was irradiated, suggesting a high DNA repair capacity during the S phase. Furthermore, we irradiated the cell nucleus in the S phase with various intensities of laser light at wavelengths of 405 nm and 375 nm and then evaluated both PCNA accumulation and the 24 h cell viability. When cells were irradiated with laser light at a wavelength of 405 nm, PCNA accumulation increased at low irradiation intensities, and almost no cell death was confirmed. Accumulation of PCNA occurred depending on the degree of DNA damage caused by laser light irradiation, suggesting appropriate DNA repair mechanisms. At high irradiation intensities, saturation of PCNA accumulation was observed, and a decrease in the cell viability was confirmed, indicating that PCNA accumulation was not sufficient for DNA repair. When exposed to laser light with a wavelength of 375 nm, PCNA accumulated in proportion to the intensity of irradiation. Despite PCNA accumulation levels similar to those induced by the irradiation with laser light of 405 nm wavelength, a decrease in cell viability was observed even at lower irradiation intensities of 375 nm laser light, suggesting an increase in phototoxicity as the wavelength shortened. By evaluating the accumulation of PCNA at the site of laser irradiation, the phototoxicity of short-wavelength laser light could be quantitatively evaluated from non-lethal low to lethal high irradiation intensity.

## Data Availability

The data presented in this study are available from the corresponding author upon request.
